# Design of High-Remanence Nd-Fe-B Hot-Pressed Magnets by Manipulating Coercivity of Hydrogenation-Disproportionation-Desorption-Recombination Treated Anisotropic Precursors

**DOI:** 10.3390/ma16247599

**Published:** 2023-12-11

**Authors:** Jae-Gyeong Yoo, Tae-Hoon Kim, Hee-Ryoung Cha, Yang-Do Kim, Jung-Goo Lee

**Affiliations:** 1 Department of Magnetic Materials, Korea Institute of Materials Science, Changwon 51508, Republic of Korea; worud1114@kims.re.kr (J.-G.Y.); chopkr@kims.re.kr (T.-H.K.); h.cha@kims.re.kr (H.-R.C.); 2 Department of Materials Science and Engineering, Pusan National University, Busan 46241, Republic of Korea

**Keywords:** Nd-Fe-B permanent magnets, HDDR powders, magnetic alignment process, hot-press process

## Abstract

We propose a method of manipulating the coercivity of anisotropic hydrogenation-disproportionation-desorption-recombination (HDDR) powders to fabricate high-remanence and fine-grained Nd-Fe-B magnets using only hot-pressing without a subsequent hot-deformation process. By reducing the Nd content of anisotropic HDDR precursors such that their coercivity (H_cj_) is lowered, the c-axis of each HDDR particle is well-aligned parallel to the direction of the applied magnetic field during the magnetic alignment step. This is because the magnetic repulsive force between adjacent particles, determined by their remanent magnetization, decreases as a result of the low coercivity of each particle. Therefore, after hot-pressing the low-H_cj_ HDDR powders, a significantly higher remanence (11.2 kG) is achieved in the bulk than that achieved by hot-pressing the high-H_cj_ HDDR powders (8.2 kG). It is clearly confirmed by the large-scale electron backscatter diffraction (EBSD) analysis that the alignment of the c-axis of each anisotropic HDDR particle in the bulk is improved when low-H_cj_ HDDR powders are used to fabricate hot-pressed magnets. This coercivity manipulation of HDDR powders can be a helpful method to expand the use of HDDR powders in fabricating anisotropic Nd-Fe-B bulk magnets.

## 1. Introduction

The Nd-Fe-B permanent magnets have become indispensable materials for high-efficiency motors and generators in hybrid/electric vehicles owing to their remarkable coercivity, H_cj_, and remanence, 4πM_r_ [1,2,3]. To address the thermal demagnetization problem of Nd-Fe-B magnets at elevated temperatures during the operation of motors, their room-temperature H_cj_ should be enhanced [4,5]. It is well known that the H_cj_ of Nd-Fe-B magnets undoubtedly improves without the use of heavy rare-earth (HRE) elements when Nd_2_Fe_14_B (2-14-1) grains are refined and magnetically decoupled by Nd-rich grain boundary phases (GBPs) [4,6,7]. The hydrogenation-disproportionation-desorption-recombination (HDDR) process is an effective method for producing anisotropic Nd-Fe-B magnetic powders with ultrafine grain sizes close to a single-domain size of 2-14-1 (~250 nm) [8,9]. However, there is a critical shortcoming that limits the use of HDDR powders in fabricating bulk magnets (HDDR magnets): after sintering of HDDR powders, the H_cj_ rapidly decreases due to substantial grain coarsening [10]. Various densification methods, such as SPS (spark plasma sintering) [11] and PLP (press-less sintering) [12], which can suppress grain growth during densification, have been attempted to fabricate HDDR magnets; however, the H_cj_ that can be achieved in HDDR magnets is only about 13 kOe because of the strong exchange coupling between neighboring 2-14-1 grains via discontinuous and ferromagnetic Nd-rich GBPs [13,14,15].

The hot-deformation process, which consists of a hot-press step and a subsequent hot-deformation step [16,17], seems to be a promising process for producing high-H_cj_ HDDR magnets because it specializes in the densification of ultrafine-grained Nd-Fe-B powders such as HDDR and melt-spun powders [18,19,20,21]. According to the current investigations [22,23], the HDDR magnets, comprising 400 ± 100 nm-sized 2-14-1 grains surrounded by more continuous Nd-rich GBPs, can be obtained by the hot-deformation of HDDR powders [22,23]. However, in this case, it is difficult to obtain a strong [001]-texture in the magnets because the grain size of the HDDR powders is not fine enough to minimize their deformation resistance, as reported by Kirchner et al. [24]. Since the HDDR powders can be fabricated into magnetically anisotropic powders [25,26], the limitation of hot-deformed HDDR magnets (i.e., low remanence due to a poor [001]-texture) can be addressed if the [001]-axis of each anisotropic HDDR powder is aligned by an external magnetic field prior to densification. In this approach, the 4πM_r_ of each HDDR powder is the most important factor for well-aligning the HDDR powders in a magnetic alignment process because the repulsive force (F) between the neighboring particles, defined as $F=\frac{P_{1}P_{2}}{d^{2}}$ (where P_1_ and P_2_ are the pole strengths of the particles and d is the distance between the particles) [27], appears when the external magnetic field vanishes at the final stage of the magnetic alignment process, as reported by Soda et al. [28]. This is because the HDDR powders are magnetized and their [001]-axes are aligned in the same direction when the HDDR powders are exposed to a strong magnetic field during the magnetic alignment process, and their magnetization is left as much as their 4πM_r_ after the external field vanishes. Thus, the higher the 4πM_r_ of the HDDR particles (P of each HDDR particle), the stronger the F between the particles, leading to more severe misalignment of the HDDR particles after the magnetic alignment step [28].

**Figure 1:**
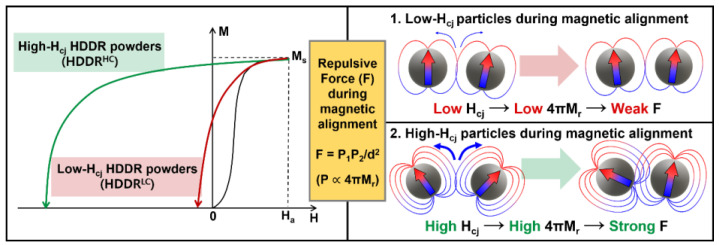
Schematic illustrations on relationship between H_cj_ of HDDR precursors and disordering of [001]-axis of HDDR powders (cases 1 and 2) during magnetic alignment process.

Therefore, the 4πM_r_ of HDDR powders should be controlled to be lower to well align the [001]-axis of each HDDR particle during the magnetic alignment step prior to the hot-press process. If a magnetic alignment process for HDDR powders is successfully developed, fine-grained anisotropic Nd-Fe-B magnets with high 4πM_r_ can be easily obtained using only a hot-press step without a subsequent hot-deformation step, which is a necessary step for obtaining a [001]-texture of the bulk in the conventional hot-deformation process [16,17]. Because the 4πM_r_ of HDDR powders can be controlled by manipulating their H_cj_ as schematically illustrated in Figure 1 [29], in this work, we attempted to fabricate high-remanence and fine-grained hot-pressed magnets by manipulating the H_cj_ of the initial anisotropic HDDR powders. To control the 4πM_r_ of HDDR powders to be lower, their H_cj_ was manipulated to be lower by decreasing the Nd content of the powders. The lower the Nd content of the powders, the stronger the exchange coupling between neighboring 2-14-1 grains, which leads to a decrease in magnetization in the early stage of the demagnetization process, as shown using a red line in Figure 1. The influences of the magnetic and microstructural properties of the initial HDDR powders on the magnetic alignment and hot-press behavior were systematically investigated. Based on the results, a guide to fabricating anisotropic HDDR magnets using hot-pressing and increasing their 4πM_r_ was proposed.

## 2. Materials and Methods

To prepare anisotropic HDDR powders with different H_cj_ values, alloys with compositions of Nd_x_Fe_87.2-x_Nb_6.6_Ga_0.6_B_5.6_ (at.%) (x = 11.8, 12.0, 12.2, and 12.5) were employed as the starting materials. The anisotropic HDDR powders were produced via the HDDR process under a condition optimized in our previous study [30], as follows: First, the powders were heated up to 840 °C under an Ar gas atmosphere with a pressure of 1.1 atm. When the temperature reached up to 840 °C, H_2_ gas flowed at a pressure of 0.3 atm for 90 min to induce an HD (hydrogenation-disproportionation) reaction. In the DR (desorption-recombination) stage, the furnace was evacuated with a rotary pump and maintained for 30 min. Finally, the powders were quenched down to room temperature using Ar gas. After the HDDR process, the particle size was in the range of 125–300 μm. The HDDR particles were filled into a graphite mold and slightly compressed using a graphite rod. At this stage, Nd_70_Cu_30_ melt-spun powders (wheel speed of 35 m/s) were mixed with the HDDR powders with x = 11.8, 12.0, and 12.2, such that the Nd content of all the final hot-pressed samples was equivalent to 12.5 at.%. To align the HDDR particles magnetically, a pulsed magnetic field of 50 kOe was applied to a mold filled with HDDR powder. Then, the green compacts were hot-pressed at 700 °C for 3 min under 400 MPa in a vacuum.

The magnetic properties of the initial HDDR powders and the final hot-pressed magnets were characterized using a vibrating sample magnetometer (VSM; VSM 7407, Lakeshore, H_max_ = 20 kOe) and a pulsed-field magnetometer (PFM; PFM14.CN, HIRST Magnetic Instruments Ltd., H_max_ = 70 kOe), respectively. The crystal structures of the samples were determined using an X-ray diffractometer (XRD; D/MAX-2500V, Rigaku, Tokyo, Japan) under Cu-K_α_ radiation. The overall microstructural observation was performed using field-emission scanning electron microscopy (FESEM; JSM-7001F, JEOL Co., Ltd., Tokyo, Japan) with an acceleration voltage of 15 kV. The crystallographic [001]-texture of the magnets was identified via electron backscatter diffraction (EBSD) using field-emission scanning electron microscopy (FESEM; JSM-7900F, JEOL Co., Ltd., Tokyo, Japan).

## 3. Results and Discussion

**Figure 2:**
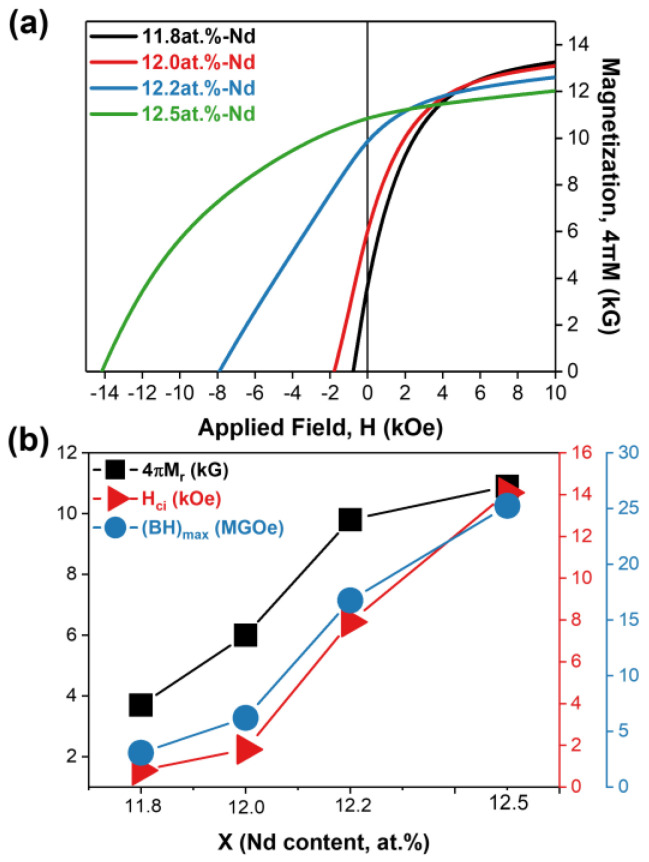
Schematic magnetic properties of initial anisotropic HDDR precursors with compositions of Nd_x_Fe_87.2-x_Nb_6.6_Ga_0.6_B_5.6_ (at.%) (x = 11.8, 12.0, 12.2, and 12.5): (**a**) demagnetization curves; (**b**) variation in the 4πM_r_, H_cj_, and (BH)_max_ values as a function of x.

**Figure 3:**
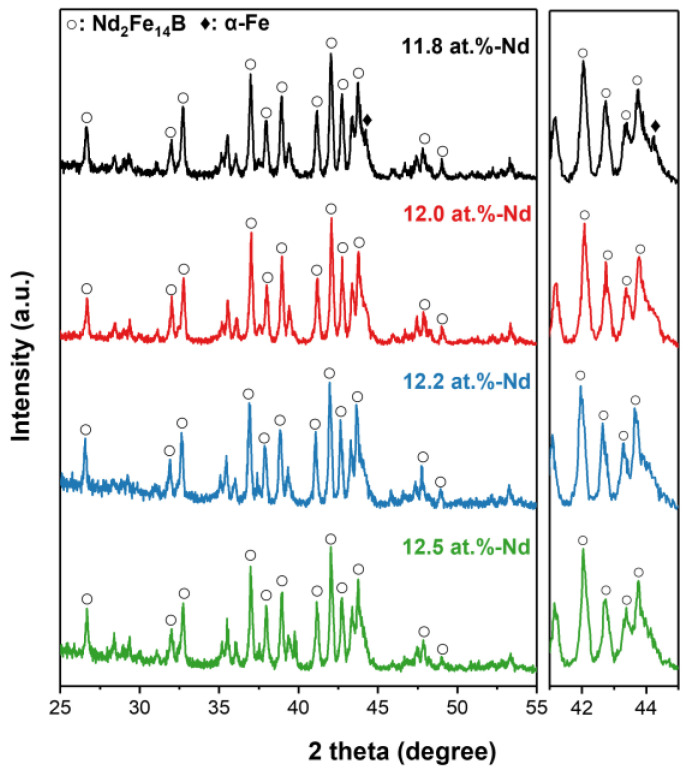
XRD patterns of initial anisotropic HDDR powders. To identify phases formed in anisotropic HDDR powders clearly, the samples for XRD analysis are pulverized into finer particles with sizes of 20 μm.

**Figure 4:**
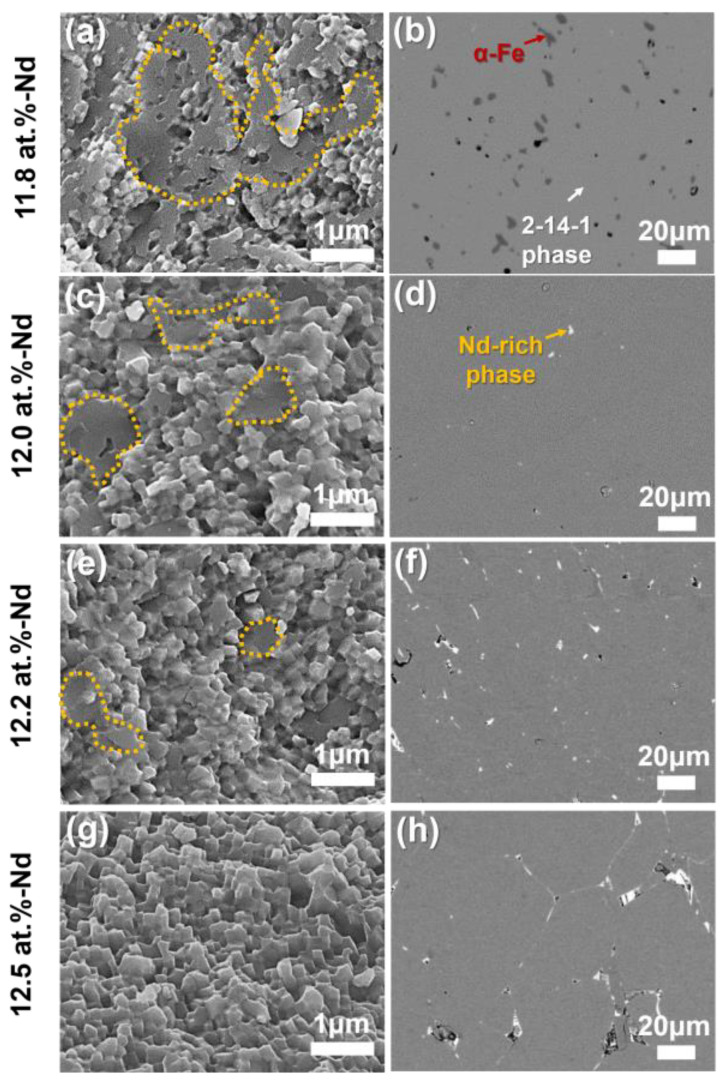
Changes in microstructure of anisotropic HDDR powders as a function of Nd content. The 1st column (**a**,**c**,**e**,**g**) represents secondary electron (SE) images taken from the fracture surface, and the 2nd column (**b**,**d**,**f**,**h**) represents back-scattered electron (BSE) images captured from the polished surface.

To evaluate the feasibility of the 4πM_r_-control of the anisotropic HDDR powders through the manipulation of their H_cj_ values, the magnetic properties of the anisotropic HDDR powders with compositions of Nd_x_Fe_87.2-x_Nb_6.6_Ga_0.6_B_5.6_ (at.%) (x = 11.8, 12.0, 12.2, and 12.5) were characterized. Figure 2a shows the demagnetization curves of the anisotropic HDDR powders containing 11.8, 12.0, 12.2, and 12.5 at.% Nd. The black, red, blue, and green solid lines correspond to the demagnetization curves for the sample with the Nd contents of 11.8, 12.0, 12.2, and 12.5 at.%, respectively. The variation in the 4πM_r_, H_cj_, and (BH)_max_ values of the anisotropic HDDR powders as a function of their Nd content can be seen in Figure 2b. As the Nd content decreases from 12.5 at.% to 11.8 at.%, the H_cj_ and 4πM_r_ of the anisotropic HDDR powder gradually decrease simultaneously. The coercivity decreases from 14 kOe to 1 kOe, and their 4πM_r_ also decreases from 11 kG to 4 kG as the Nd content decreases from 12.5 at.% to 11.8 at.%, as shown in Figure 2b. This result directly indicates that the 4πM_r_ of powders can be reduced by controlling their H_cj_ to be lower. To confirm the constituent phases and their crystal structure in the anisotropic HDDR powders, X-ray diffraction (XRD) analysis was performed. Figure 3 shows the XRD patterns of the anisotropic HDDR powders with compositions of Nd_x_Fe_87.2-x_Nb_6.6_Ga_0.6_B_5.6_ (at.%) (x = 11.8, 12.0, 12.2, and 12.5). In the XRD patterns, the 11.8, 12.0, 12.2, and 12.5 at.% Nd-containing anisotropic HDDR powders correspond to the black, red, blue, and green solid lines in the first, second, third, and fourth rows in Figure 3, respectively. As shown in Figure 3, distinct diffraction peaks from the 2-14-1 main phase (tetragonal P42/mnm, a = b = 0.88050 nm, c = 1.22050 nm) are observed in all the samples (indexed with empty circles in Figure 3). In the case of the 11.8 at.% Nd-containing HDDR powders (1st row of Figure 3), a weak diffraction peak from the α-Fe phase (cubic, a = 0.28665 nm) appears. According to the pseudobinary phase diagram of the Nd-Fe-B system [8], the α-Fe phase can be stabilized when the Nd content of the magnets is low and their composition is close to the stoichiometry of 2-14-1 [8]. In order to confirm the formation of the phases and their distribution within the samples, the SEM analysis was conducted as shown in Figure 4.

Figure 4 shows the changes in the microstructure of the anisotropic HDDR powders as a function of their Nd content. The 1st column (Figure 4a,c,e,g) presents secondary electron (SE) images taken from the fracture surface, and the 2nd column (Figure 4b,d,f,h) presents backscattered electron (BSE) images taken from the polished surface. The SE images of the fracture surface reveal the average grain size and the distribution of the Nd-rich GBPs, and the BSE images of the polished surface reveal the constituent phases of the samples. As shown in Figure 4g, 2-14-1 grains with sizes of approximately 300 nm were homogeneously formed in the 12.5 at.% Nd-containing HDDR powders. Because the fracture of Nd-Fe-B alloys occurs along the Nd-rich GBPs [31], the homogeneous morphology of the 2-14-1 grain on the fracture surface directly indicates that uniform and continuous Nd-rich GBPs are formed within the samples [31,32]. Therefore, in the 12.5 at.% Nd-containing HDDR powders, the Nd-rich GBPs are also expected to be uniformly formed. However, in contrast to the 12.5 at.% Nd-containing HDDR powders, the unfractured region is shown in Figure 4a,c,e, marked by the yellow dotted area. This indicates that the Nd-rich GBP-free regions appear in the low-Nd samples at x = 11.8, 12.0, and 12.2, and the areal fraction of such regions increases with decreasing Nd content of the HDDR powder. In addition, the formation of the α–Fe phase in the 11.8 at.% Nd-containing HDDR powdes can be clearly seen in the BSE-SEM image of Figure 4. The three kinds of phases with distinct contrasts of black, gray, and white are observed in the BSE-SEM images, and those phases correspond, respectively, to the α–Fe phase, the Nd_2_Fe_14_B main phase, and the Nd-rich phase, indicated by red, white, and yellow arrows. The black contrast α–Fe phase is observed only in the 11.8 at.% Nd-containing HDDR powders, which is consistent with the results from the XRD analysis shown in Figure 3. From the BSE images in Figure 4b,d,f,h showing the Nd-rich phase, it can be expected that the reason for the formation of no Nd-rich GBP region in the lower Nd content HDDR powders is that the excess Nd involved in the formation of the Nd-rich GBP becomes less with decreasing Nd content in the powders [33,34]. As a result, the exchange coupling between adjacent 2-14-1 grains becomes stronger, and thus, the H_cj_ of the HDDR powders decreases with decreasing Nd content, as shown in Figure 2. Note that the 4πM_r_ of the HDDR powders also decreases with a decrease in their H_cj_ value, as shown in Figure 2b. This is because the lower the H_cj_ of the HDDR powders, the weaker their resistance to magnetization changes; thus, demagnetization starts earlier, as shown in Figure 2a [4]. For this reason, by manipulating the H_cj_ of HDDR powders, their 4πM_r_ can also be easily controlled, as reported by Li, W.F. et al. [29]. Except for the 11.8 at.% Nd-containing HDDR powders, no secondary phases were observed in the 12.0, 12.2, and 12.5 at.% Nd-containing HDDR powders, as shown in Figure 3 and Figure 4, which indicates that the HDDR reactions are ideally completed in the samples with the Nd content of 12.0, 12.2, and 12.5 at.%. Based on the results from the magnetic and microstructural characterizations of the initial HDDR powder, we adopted the 12.0 at.% and 12.5 at.% Nd-containing powders as low- and high-H_cj_ HDDR powders for the fabrication of fine-grained hot-pressed bulks, respectively, to compare the magnetic properties and [001]-texture after the hot-press of the low- and high-H_cj_ HDDR powders. Note that a pulsed magnetic field of 50 kOe was applied to the anisotropic HDDR powders to align their [001]-axes prior to densification using a hot-press process. Also, because the magnetic properties of Nd-Fe-B bulks are significantly dependent on their Nd content, the Nd_70_Cu_30_ melt-spun powders were mixed with the low-H_cj_ HDDR powders before the magnetic alignment step, such that both the final hot-pressed samples fabricated from the low- and high-H_cj_ HDDR powders contained the same Nd contents of 12.5 at.%. The difference in the Cu contents between the final hot-pressed samples induced by adding the Nd_70_Cu_30_ melt-spun powders was negligible (~0.2 at.%). Hereafter, the low- and high-H_cj_ HDDR powders are referred to as HDDR^LC^ and HDDR^HC^, respectively, and the hot-pressed magnets fabricated from the magnetically aligned low- and high-H_cj_ HDDR powders are referred to as HP-HDDR^LC^ and HP-HDDR^HC^, respectively.

**Figure 5:**
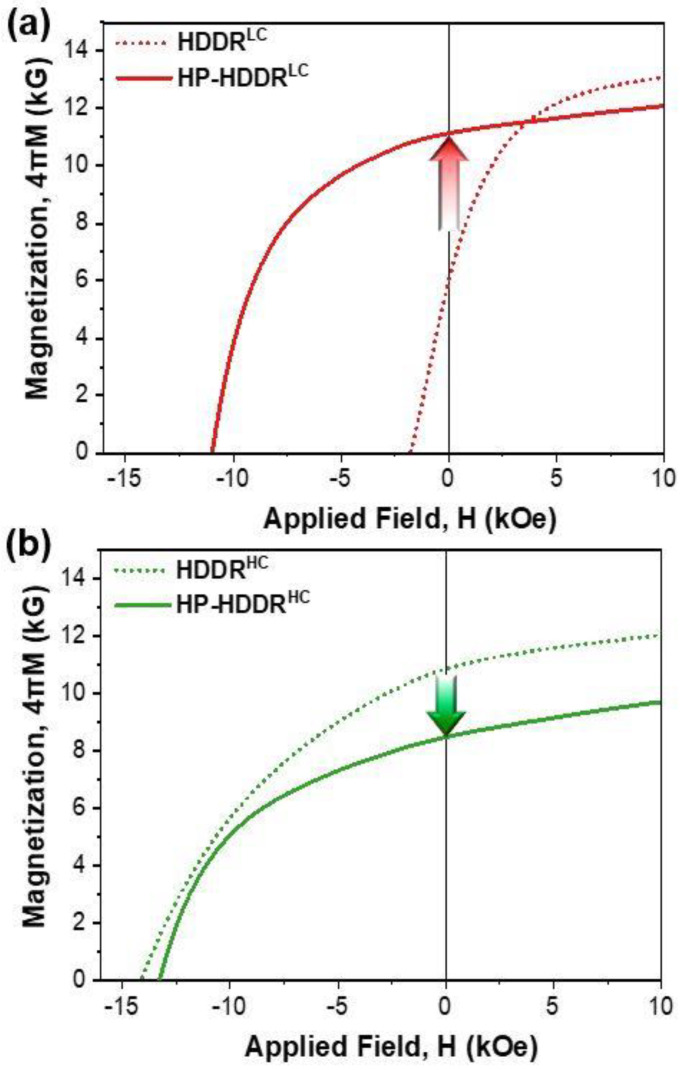
Magnetic properties of hot-pressed magnets fabricated from (**a**) low-H_cj_ and (**b**) high-H_cj_ HDDR precursors (solid lines). Dotted lines displayed in (**a**,**b**) correspond to the demagnetization curves for low-H_cj_ and high-H_cj_ HDDR precursors, respectively.

**Figure 6:**
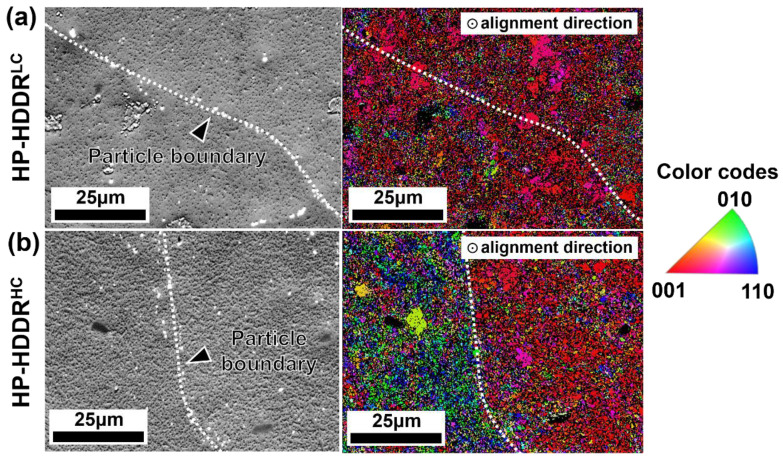
EBSD inverse pole figures (IPF) taken from the particle boundary of HDDR powders in hot-pressed magnets fabricated from (**a**) low-H_cj_ and (**b**) high-H_cj_ HDDR precursors. The alignment direction of HDDR powders is out-of-plane in the images.

Figure 5 shows the demagnetization curves of hot-pressed magnets fabricated from the magnetically aligned low- and high-H_cj_ HDDR powders. The solid lines in Figure 5a,b correspond to the HP-HDDR^LC^ and HP-HDDR^HC^ magnets, respectively, and the dotted lines in Figure 5a,b correspond to the HDDR^LC^ and HDDR^HC^ precursors, respectively. After magnetic alignment and hot-pressing, the 4πM_r_ of the HDDR^LC^ precursors increases from 6 kG to 11.2 kG, whereas that of the HDDR^HC^ precursors decreases from 11 kG to 8.2 kG, as shown in Figure 5. It should be noted that the 4πM_r_ obtained in the final hot-pressed magnets is significantly higher when the HDDR^LC^ powders are magnetically aligned and hot-pressed. The density and Nd content, which are among the primary factors affecting the 4πM_r_ of the magnets [35], were the same in the HP-HDDR^LC^ and HP-HDDR^HC^ magnets (density = ~98.7% and Nd content = 12.5 at.%). This implies that the [001]-axis alignment of HDDR particles in the HP-HDDR^LC^ magnets is much higher than that in the HP-HDDR^HC^ magnets. In order to confirm the alignment of each of the HDDR powders in the HP-HDDR^LC^ magnets and HP-HDDR^LC^ magnets clearly, a large-scale electron backscatter diffraction (EBSD) was performed as shown in Figure 6.

Figure 6 shows the EBSD inverse pole figure (IPF) maps of (a) the HP-HDDR^LC^ and (b) the HP-HDDR^HC^ magnets observed along the alignment direction. To clearly observe the difference in the [001]-axis alignment of each anisotropic HDDR particle between the HP-HDDR^LC^ and HP-HDDR^HC^ magnets, a large-scale EBSD analysis was conducted at the particle boundary region. It can be clearly seen in Figure 6 that the [001]-axes of the HDDR powders in the HP-HDDR^LC^ magnets are well aligned, whereas those in the HP-HDDR^HC^ magnets are misoriented. Therefore, it can be concluded that the higher 4πM_r_ of the HP-HDDR^LC^ magnets shown in Figure 5 is attributed to the higher [001]-axis alignment of the HDDR^LC^ powders during the magnetic alignment step. Why, then, are the HDDR^LC^ powders well-aligned in the magnetic alignment process? According to a previous report [28], during the magnetic alignment of anisotropic HDDR powders, a magnetic repulsive force, which induces disordering of the [001]-axis of each particle, can be generated between neighboring particles because of their 4πM_r_. This is because the HDDR particles are magnetized, and their [001]-axes are aligned in the same direction when exposed to a strong external field at the early stage of the magnetic alignment process, and their magnetization is left by their 4πM_r_ even after the external magnetic field vanishes at the final stage of the magnetic alignment process. As the magnetic repulsive force (F) between neighboring magnetic particles with pole strengths of P_1_ and P_2_ is defined as $F=\frac{P_{1}P_{2}}{d^{2}}$ [27], HDDR powders with low-4πM_r_ (i.e., low-P) are advantageous for reducing the magnetic repulsive force between neighboring particles whose [001]-axes are aligned in the same direction. Because the 4πM_r_ of HDDR powders can be lowered by decreasing their H_cj_, as shown in Figure 1 and Figure 2, anisotropic hot-pressed magnets with higher 4πM_r_ and stronger [001]-texture can be obtained after magnetic alignment and hot-pressing of the HDDR powders by manipulating their H_cj_, as shown in Figure 5 and Figure 6.

**Figure 7:**
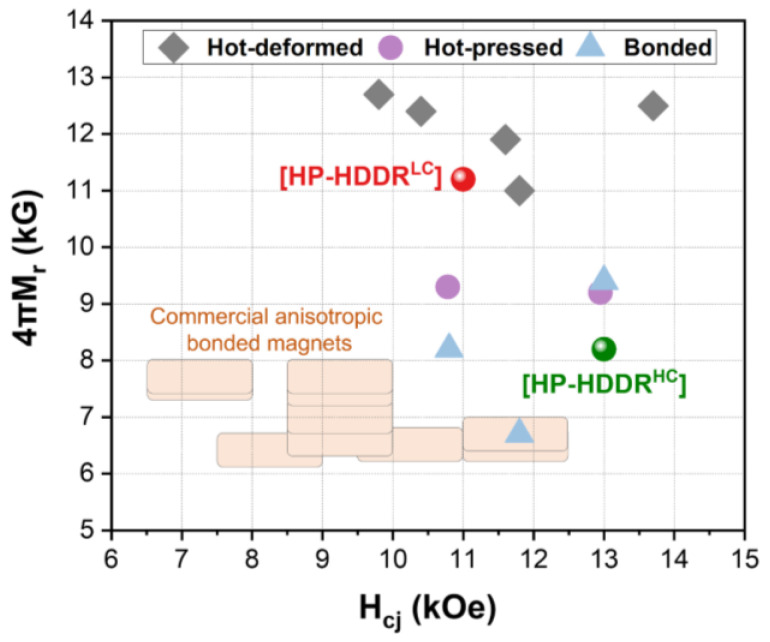
H_cj_ and 4πM_r_ values of hot-pressed, bonded, and hot-deformed HDDR magnets extracted from [15,21,22,23,36,37,38,39]. To verify the magnetic performance of the magnets developed in this work, the H_cj_ and 4πM_r_ values of our hot-pressed magnets are displayed using red and green spheres.

To verify the magnetic performance of the magnets developed in this work, the H_cj_ and 4πM_r_ of the HP-HDDR^LC^ and HP-HDDR^HC^ magnets are compared with those of Nd-Fe-B hot-pressed, hot-deformed, and bonded magnets produced from anisotropic HDDR powders reported in other studies [15,21,22,23,36,37,38,39], as shown in Figure 7. The hot-pressed magnets developed in this work (HP-HDDR^LC^) exhibited higher 4πM_r_ than those of the hot-pressed and bonded magnets but showed lower 4πM_r_ than those of the hot-deformed magnets reported in other studies. Although the 4πM_r_ of the HP-HDDR^LC^ magnets was lower than that of hot-deformed magnets produced from HDDR powders, as shown in Figure 7, the fabrication method for the HP-HDDR^LC^ magnets was significant in that anisotropic bulk magnets with higher 4πM_r_ could be successfully fabricated using only hot-pressing without undergoing the subsequent hot-deformation process. In conclusion, by decreasing the H_cj_ of anisotropic HDDR precursors, their 4πM_r_ value, which determines the strength of the repulsive force between adjacent particles during magnetic alignment, was successfully lowered as shown in Figure 2, Figure 3 and Figure 4, thus the anisotropic bulk magnets with stronger [001]-texture and higher 4πM_r_ could be obtained via the magnetic alignment and hot-pressing of the HDDR^LC^ powders as shown in Figure 5 and Figure 6.

## 4. Conclusions

In this work, we proposed a novel guide for fabricating anisotropic Nd-Fe-B magnets via the hot-pressing of HDDR powders. We demonstrated that the 4πM_r_ of HDDR precursors should be decreased to well-align the [001]-axis of each HDDR powder during the magnetic alignment step. By lowering the 4πM_r_ of the HDDR precursors, the magnetic repulsive force between the precursors, inducing the disordering of the [001]-axis of the precursors, becomes weaker. It was also demonstrated that the 4πM_r_ of the HDDR precursors could be easily decreased by decreasing their Nd content, such that the Hcj of the HDDR precursors became lower. Therefore, by using precursors with low-H_cj_ (i.e., low 4πM_r_ and low Nd content) in fabricating anisotropic hot-pressed bulk magnets, significantly higher remanence (11.2 kG) and stronger [001]-texture can be obtained in the final hot-pressed magnets than those of anisotropic hot-pressed bulk magnets prepared using high-H_cj_ HDDR precursors. We believe that the fabrication method of anisotropic hot-pressed magnets proposed in this work is helpful to the extensive use of HDDR powders in the fabrication of anisotropic Nd-Fe-B magnet bulks.

## Data Availability

The data presented in this research are available on request from the corresponding authors.
